# Polymeric Nanocapsules Containing Kojic Acid Dipalmitate and Rosehip Oil: Development and Evaluation of Preliminary Efficacy and Safety for Skin Whitening

**DOI:** 10.1021/acsomega.5c08878

**Published:** 2025-12-12

**Authors:** Júlia Capp Zilles, Marya Alexandrina Vallenot Lemos, Larissa Pedron Duarte, Maria Paula Faccin Huth, Irene Clemes Kulkamp Guerreiro, Bonnie C. Carney, Aline Rigon Zimmer, Renata Vidor Contri

**Affiliations:** † Programa de Pós-Graduação em Ciências Farmacêuticas, 28124 Universidade Federal do Rio Grande do Sul, Porto Alegre, Rio Grande do Sul 90610-000, Brazil; ‡ Faculdade de Farmácia, Universidade Federal do Rio Grande do Sul, Porto Alegre, Rio Grande do Sul 90610-000, Brazil; § Firefighters’ Burn and Surgical Research Laboratory, MedStar Health Research Institute, Washington, District of Columbia 20010, United States; ∥ School of Medicine, Department of Biochemistry and Molecular & Cellular Biology, Georgetown University, Washington, District of Columbia 20007, United States

## Abstract

Polymeric nanocapsules associated with kojic acid dipalmitate (KDP) and rosehip oil can be a strategy to obtain high-performance skin whitening/lightning formulations. Nanocapsules containing 0.1% of KDP and 5% of rosehip oil were developed with different polymers (Eudragit RS100, Eudragit S100, poly­(ε-caprolactone) (PCL) and poly­(d,l-lactide-*co*-glycolide) (PLGA)) to limit the contact of KDP with the external media and avoid its degradation. The nanocapsules had a diameter of less than 200 nm, with suitable size distribution, a pH range between 2.5 and 5.0, a KDP content of 0.9 mg/mL and an encapsulation efficiency above 99%. The zeta potential was positive for the Eudragit RS100 nanocapsules and negative for the other nanocapsules. The PLGA nanocapsules were selected due to better stability results, since there was no decay in KDP content after 180 days (refrigerated storage). The in vitro skin permeation assay showed that KDP reaches the epidermis. This formulation showed in vitro antioxidant activity through both the DPPH assay and the β-carotene/linoleic acid assay when used at 25 μg/mL of KDP, with higher activity than a kojic acid solution (KDP correspondent concentration) and, at 13.75 μg/mL, inhibited tyrosinase in vitro to 17%, significantly greater than the kojic acid solution. A 25% melanin reduction was obtained after normal human epidermal melanocytes (NHEM) were treated with 3.1 μg/mL of KDP. Cytocompatibility was observed after 24 h treatment with up to 6.25 μg/mL of the formulation in fibroblast-like cells (3T3-L1) and up to 3.1 μg/mL in NHEM. Therefore, PLGA nanocapsules containing KDP and rosehip oil have potential for use in skin whitening/lightning formulations.

## Graphical abstract

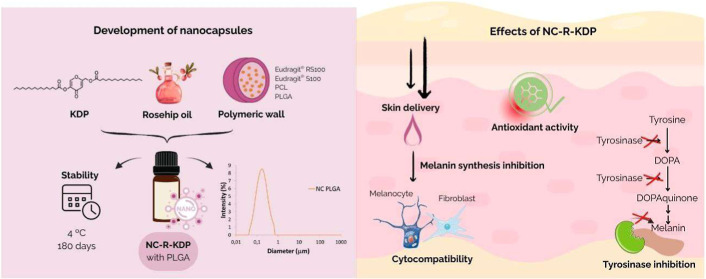

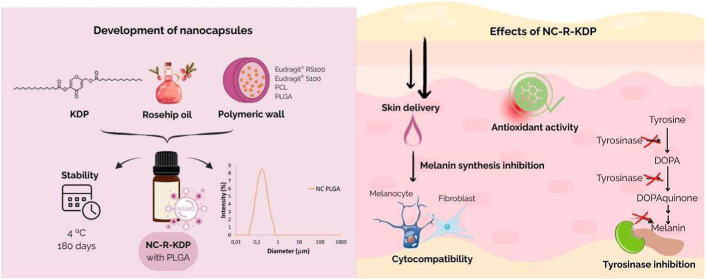

## 1 Introduction

The interest in developing cosmetic formulations containing effective and safe bioactive compounds has increased, especially in regards to their stability, skin penetration and skin compatibility.^1^ Kojic acid is an organic acid derived from fungi fermentation, such as *Aspergillum* and *Penicillium*, and it is widely studied for its skin whitening properties and its capacity to inhibit tyrosinase, the key enzyme in melanin synthesis.^2^ However, its use is limited because of its low stability, sensitivity to light and heat, and oxidation. Additionally, adverse effects such as contact dermatitis and skin irritation have been reported.^3–5^

To overcome the limitations of kojic acid, kojic acid derivatives, such as kojic acid dipalmitate (KDP), appear to be promising alternatives. KDP is the esterified form of kojic acid, and it is stable to light and heat and in a wide pH range without suffering color alterations.^6,7^ The KDP molecule is hydrolyzed by esterases located on the skin, releasing kojic acid in situ.^6^ Due to KDP’s lipophilic characteristics, it may have increased affinity with the stratum corneum and thus greater cutaneous absorption.^3–5^ KDP remains difficult to incorporate into aqueous formulations.

Rosehip oil is extracted from *Rosa* spp. seeds and is known for its skin regenerative properties, antioxidant, anti-inflammatory and skin whitening activities.^8,9^ A serum containing rosehip oil was tested in a single-arm, open-label study and showed reduction in skin pigmentation in a time dependent manner, highlighting the potential of rosehip oil as a skin whitening agent.^10^ This oil is rich in unsaturated fatty acids, phenolic compounds and transretinoic acid.^8,11,12^ However, rosehip oil is easily oxidized.^12^

Nanotechnology has become an important technique in the development of high-performance and high-stability cosmetic formulations. Nanocarriers can be used to incorporate bioactive compounds, leading to a targeted delivery of active substances, improving skin penetration and protecting the active ingredients.^13^ Polymeric nanocapsules are vesicular systems constituted by a polymeric wall that surrounds an aqueous or oily core. This type of nanocarrier can be used to incorporate lipophilic active substances, leading to protection of the active substances against chemical and physical degradation, to a controlled release of the substances, and to enhanced solubility, enabling their incorporation into aqueous vehicles.^14,15^ Polymers such as Eudragit RS100, Eudragit S100, poly­(ε-caprolactone) (PCL) and poly­(d,l-lactide-*co*-glycolide) (PLGA) are frequently used in the synthesis of nanocapsules due to their barrier function and biocompatibility.^12,14,16–18^

The association of KDP and rosehip oil within a nanocarrier represents a promising strategy for the development of innovative cosmetic formulations with potential to overcome challenges related to stability and skin permeation. An oil-in-water nanoemulsion associating these two active ingredients has already been developed by our research group.^11^ However, this system showed limited stability, as KDP underwent hydrolysis in the aqueous environment.^11^ Other nanotechnological systems have already been proposed for the delivery of KDP, including nanoemulsions,^11,19,20^ multiple emulsions,^6^ liposomes,^21^ ethosomes^7^ and solid lipid nanoparticles.^22^ To the best of our knowledge, polymeric nanocapsules containing KDP have not yet been described, and this nanocarrier may therefore represent a more stable and protective alternative for KDP delivery, limiting its contact with aqueous medium.

In view of the above, this investigation aimed at associating KDP and rosehip oil into polymeric nanocapsules, in order to obtain a high-performance and high-stability formulation. Different polymers were tested. The most promising formulation in terms of stability was also assayed regarding skin permeation, antioxidant and skin whitening performance, and cytocompatibility with skin cells.

## 2 Materials and Methods

### 2.1 Materials

KDP was purchased from SM Empreendimentos Farmacêuticos Ltd.a. (São Paulo, Brazil), rosehip oil and caprylic/capric triglycerides were obtained from Delaware LTA (Porto Alegre, Brazil), and polysorbate 80 was obtained from Labsynth (Diadema, Brazil). Eudragit RS100 was kindly donated by Evonik (Essen, Germany), Eudragit S100 was obtained from Almapal S/A (São Paulo, Brazil), Poly­(d,l-lactide-*co*-glycolide) 75/25 was obtained from Corbion (Amsterdam, Netherlands), and poly­(ε-caprolactone) (number-average molecular mass, Mn 80,000) was obtained from Perstorp UK Limited (Warrington, England). Tetrahydrofuran (THF) was purchased from Química Moderna (Barueri, Brazil), and acetonitrile was purchased from J.T. Baker (Phillipsburg, NJ, USA). Methanol, 2,2-diphenyl-1-picrylhydrazyl (DPPH), betacarotene, linoleic acid and sorbitan monostearate were obtained from Sigma-Aldrich Brasil Ltd.a. (Cotia, Brazil). Tyrosinase (100 kU, 1560 U/mg, Worthington) was purchased from Sinapse biotecnologia (São Paulo, Brazil). The 3T3-L1 cell line was purchased from the Rio de Janeiro Cell Bank (BCRJ), Brazil. Fetal bovine serum (FBS) was purchased from Cripion (Andradina, SP, BR); phosphate-buffered saline (PBS) was purchased from Laborclin (São José do Rio Preto, SP, BR). Dulbecco’s Modified Eagle Medium (DMEM), 3-(4,5-dimethylthiazol-2-yl)-2,5-diphenyltetrazolium bromide (MTT), synthetic melanin, PBS, dimethyl sulfoxide (DMSO), penicillin/streptomycin, amphotericin, trypsin, and other reagents for cell culture were obtained from Sigma-Aldrich (St. Louis, MO, USA). All other chemicals or reagents were of analytical grade and used as received.

### 2.2 Development of Nanocapsules

Nanocapsules containing 0.1% of kojic acid dipalmitate (KDP) and 5% of rosehip oil were developed through interfacial deposition of preformed polymer method, a bottom-up technique.^23^ The formulation was produced following the methodology described by Contri et al.^12^ 2% of each of the following polymers were tested: Eudragit RS100, Eudragit S100, poly­(ε-caprolactone) (PCL) and poly­(d,l-lactide-*co*-glycolide) (PLGA), and the formulations were named NC ERS100, NC ES100, NC PCL and NC PLGA, respectively. An organic phase composed of KDP, rosehip oil, polymer and 100 mL of acetone (and for NC PCL and NC PLGA, 80 mg of sorbitan monostearate was also added), was kept under magnetic stirring at 60 °C and injected into an aqueous phase composed of 152 mg of polysorbate 80 and 106 mL of purified water, also kept under magnetic stirring. The acetone and part of the water were evaporated on a rotary evaporator (Rotary evaporator mod.801, Fisatom, São Paulo, Brazil) until a final volume of 10 mL was achieved. After stability tests, the nanocapsule with PLGA was named NC-R-KDP. Table 1 summarizes all the nanocapsules suspensions developed in the present study.

**Table 1:** Components of Nanocapsules Suspensions^a^

	NC ERS100	NC ES100	NC PCL	NC PLGA/NC-R-KDP	NC-R	NC-T-KDP	NC-T
water	91.4%	91.4%	90.6%	90.6%	90.7%	90.6%	90.7%
polysorbate 80	1.5%	1.5%	1.5%	1.5%	1.5%	1.5%	1.5%
KDP	0.1%	0.1%	0.1%	0.1%	-	0.1%	-
rosehip oil	5%	5%	5%	5%	5%	-	-
caprylic/capric triglyceride	-	-	-	-	-	5%	5%
eudragit RS100	2%	-	-	-	-	-	
eudragit S100	-	2%	-	-	-	-	-
PCL	-	-	2%	-	-	-	-
PLGA	-	-		2%	2%	2%	2%
sorbitan monostearate	-	-	0.8%	0.8%	0.8%	0.8%	0.8%

a KDP = kojic acid dipalmitate; PCL = poly­(ε-caprolactone) (Mn 80,000); PLGA = poly­(d,l-lactide-*co*-glycolide) 75/25; NC-R = PLGA nanocapsules with rosehip oil and without KDP; NC-T-KDP = PLGA nanocapsules with caprylic/capric triglycerides instead of rosehip oil; NC-T = PLGA nanocapsules with caprylic/capric triglycerides instead of rosehip oil and without KDP.

For comparison purposes regarding antioxidant and skin whitening activities, unloaded nanocapsules were prepared following the same protocol, with PLGA (the selected polymer after stability assays) but without KDP in the organic phase (NC-R), or with rosehip oil changed to caprylic/capric triglyceride (inert oil) (NC-T-KDP with KDP and NC-T without KDP). A kojic acid aqueous solution of 0.23 mg/mL (SOL KA) and a KDP dispersion were also prepared (D KDP). For the latter, 10 mg of KDP was mixed with 375 mg of sorbitan oleate and 375 mg of polysorbate 80 and heated until a suitable dispersion of the active substance was obtained. Then, water was slowly and constantly poured into the mixture, and the formulation was vortexed (VX-18, IONLAB, Araucária, Brazil) for 5 min.

### 2.3 Characterization of Nanocapsules

Nanocapsules were characterized in terms of their appearance, pH, mean droplet size and size distribution, zeta potential, KDP content, and KDP incorporation efficiency. The measurements were performed in triplicate of batches immediately after preparation.

pH determination was performed using a potentiometric method (Digimed, São Paulo, Brazil) at room temperature. The mean droplet size and size distribution were analyzed via two complementary techniques: laser diffraction (LD) (Malvern 2000 Mastersizer, Malvern Instruments, UK) and dynamic light scattering (DLS) (Zetasizer Nano Series, model ZEN 3600, Malvern Instruments, UK). For DLS analysis, the samples were diluted 500 times in purified water. For both LD and DLS, the refraction index was selected according to each polymer (1.38 for NC ERS100, 1.39 for NC ES100, 1.46 for NC PCL and 1.47 for NC PLGA). The electrophoretic mobility technique (Zetasizer Nano Series, model ZEN 3600, Malvern Instruments, UK) was employed to measure the zeta potential of the formulations, with samples diluted 500 times in 10 mM NaCl solution.

For the determination of KDP content and encapsulation efficiency, a high-performance liquid chromatograph coupled with an ultraviolet detector (HPLC-UV) method was employed. The analysis was carried out according to Tazesh et al. with modifications.^24^ An HPLC-UV system (Series 200, PerkinElmer, Waltham, MA, USA) and a Restek C18 column (ROC C18, 5 μm, 4.6 × 250 mm, Restek, United States) were used. The mobile phase consisted of tetrahydrofuran (THF), acetonitrile, methanol, purified water, and acetic acid (35:30:29:5:1), and the flow rate used was 1.0 mL/min, while the wavelength was set at 250 nm. The nanocapsule samples were dissolved in the same solvent mixture as the mobile phase and kept in an ultrasonic bath for 10 min for the KPD content assay. KDP encapsulation efficiency was analyzed using ultrafiltration–centrifugation (Amicon 10,000 MW, Millipore), with centrifugation performed at 5000 rpm (3 cycles of 10 min each). The KDP content in the ultrafiltrate (UF) was determined by HPLC-UV and the encapsulation efficiency was determined via eq 1

1 $$EE=\frac{\left(total\;drug\;content-UF\;drug\;content\right)}{total\;drug\;content}\times 100$$

### 2.4 Nanocapsules Stability

The stability of the developed nanocapsules was analyzed throughout 6 months, with analysis at days 0, 30, 90, and 180. The formulations were stored in amber glass flasks at refrigerated temperature (4 °C) and room temperature (25 °C). At the previously mentioned time intervals, the samples were evaluated for their appearance, pH, particle size, size distribution and KDP content. The immobilized nanocapsule suspensions were stored at 25 °C, and the samples were collected after 180 days without being shaken prior to sampling to investigate the presence of KDP crystals in the formulation.^25^ The samples were submitted to HPLC-UV analysis of KDP content as previously described. KDP crystals were considered to be present in the suspension if the KDP content was lower than the content measured for the shaken suspension.

The kojic acid content was also analyzed by HPLC-UV, as kojic acid dipalmitate can undergo hydrolysis, turning into kojic acid.^11^ The analysis was carried out according to Chang et al. with modifications.^26^ An HPLC-UV system (Series 200, PerkinElmer, Waltham, MA, USA) and a Restek C18 column (ROC C18, 5 μm, 4.6 × 250 mm, Restek, United States) were used. The mobile phase consisted of acetonitrile, purified water, and triethylamine (25:74.9:0.1), with the pH adjusted to 3.5–4.5 with acetic acid. The wavelength was set to 262 nm, and the flow rate was 0.5 mL/min. Nanocapsules samples were initially dissolved in 2.5 mL of acetonitrile, and then, purified water was added (to complete 10 mL) and kept in an ultrasonic bath for 10 min.

### 2.5 HPLC-UV Methods Validation

HPLC-UV method validation was performed for both KDP and KA. Linearity, specificity, precision (intra- and interday), and accuracy were evaluated according to the International Conference on Harmonization (ICH) guidelines Q2 (R1) for the validation of analytical methods.^27^

Linearity was evaluated through the measurement of three different calibration curves in three different days. For the KDP linearity, a standard solution was prepared in THF and diluted to the final concentrations of 1, 5, 10, 20, and 30 μg/mL (in the mobile phase), whereas for KA linearity, a standard solution of KA was prepared in 75:25 purified water:acetonitrile and diluted to the final concentrations of 1, 5, 10, 15, 20, 30, and 50 μg/mL (in 75:25 purified water:acetonitrile). The three calibration curves for each method were used to plot the average curves, and the equations were determined via linear regression. Unloaded nanocapsules were used for specificity assessment of both methods. The intraday precision (repeatability) was analyzed in 6 replicate solutions prepared individually at a concentration of 10 μg/mL (KDP) or 15 μg/mL (KA). Interday (intermediate) precision was determined by taking a total of 9 measurements within the linear interval of the method (10 μg/mL for KDP or 15 μg/mL for KA) in 3 different days (3 replicate measurements per day). The accuracy was analyzed by adding unloaded nanocapsules to KDP or KA dilutions in three different concentrations, low, medium and high, with replicates at each level (8 μg/mL, 10 μg/mL and 12 μg/mL for KDP and 5 μg/mL, 15 μg/mL and 30 μg/mL for KA). Since KA is a conversion product of KDP, the quantification limit was also assessed for the KA method, and it was calculated by multiplying 10 times the standard deviation of the response and dividing it by the slope of the calibration curve.^28^

### 2.6 In Vitro Skin Permeation

Skin permeation studies were conducted as previously described by Zilles et al.,^11^ using porcine ears as membranes (skin thickness of 1.82 ± 0.05 mm) in Franz diffusion cells. The study was performed in an infinite dose regimen (1 mL of each sample applied to the donor compartment), with 6 mL of receptor medium (7.5% polysorbate 80 aqueous solution) kept at 32 ± 1 °C under magnetic stirring. After 12 or 24 h of sample contact with the membrane (NC-R-KDP or D-KDP), aliquots of the receptor medium were collected and subjected to HPLC-UV analysis as previously described. The amount of KDP retained on different skin layers was then analyzed. The membrane was removed from the cell, and the excess of the formulation was removed with one piece of tape.^29^ The tape-stripping technique (29 tapes total) was used to remove the stratum corneum. The tapes were placed in a tube with extraction solution (THF, acetonitrile, methanol, purified water, and acetic acid at a 35:30:29:5:1 ratio). Afterward, the membrane was placed in a water bath at 60 °C for 45 s to loosen the dermo-epidermal junction and allow for the separation of the epidermis from the dermis.^11,29,30^ The epidermis was removed with a scalpel by gently scraping it, and the dermis was cut into small pieces. The samples were placed in different tubes containing extraction solution. The tubes were vortexed for 2 min, followed by 15 min in an ultrasonic bath, and then submitted to HPLC-UV analysis as previously described. This assay was performed in quadruplicate.

### 2.7 Antioxidant Activity

The antioxidant activities of the PLGA nanocapsules with rosehip oil, with or without KDP (NC-R-KDP and NC-R, respectively), of the PLGA nanocapsules without rosehip oil, with or without KDP (NC-T-KDP and NC-T, respectively), and the KA solution (SOL-KA) were investigated via two different in vitro antioxidant assays: the DPPH (2,2-diphenyl-1-picrylhydrazyl) assay^31^ and the β-carotene/Linoleic acid assay.^32^ Methanol was used as a negative control in both assays. The assays were performed in triplicate.

For the DPPH assay, 500 μL of sample were added to 1.5 mL of DPPH solution in methanol (20 μg/mL) and left in the dark for 2 h. Afterward, the samples were centrifuged (5000 rpm, 2 min), and the absorbance was measured using a spectrophotometer (K37-UVVIS, KASVI, Pinhais, PR, Brazil) at 515 nm. The free radical scavenging activity was calculated via eq 2

2 $$\%\;inhibition\;DPPH=\left[\frac{\left(A_{c}-sample\;absorbance\right)}{A_{c}}\right]\times 100$$

where *A*_c_ is the absorbance of the negative control.

For the β-carotene/Linoleic acid assay, a solution of 2 mg of β-carotene in 10 mL of chloroform was mixed with 20 mg of linoleic acid and 200 mg of polysorbate 80. The chloroform was removed by evaporation (Rotary evaporator mod.801, Fisatom, São Paulo, Brazil). Then, 50 mL of purified water saturated with oxygen was added to the mixture under constant stirring. A total of 2.5 mL of this solution was added to test tubes, followed by the addition of 200 μL of the samples (diluted 3 times in methanol). The absorbance was measured immediately after sample preparation using a spectrophotometer at 470 nm (K37-UVVIS, KASVI, Pinhais, PR, Brazil), after which the samples were incubated at 50 °C and exposed to light for 1 h for the oxidation reaction to occur. A second measurement of absorbance was performed. Samples without β-carotene were used as blanks. The antioxidant activity was calculated via eq 3

3 $$\%\;antioxidant\;activity=\left[1-\left(\frac{A_{0}-A}{A_{c0}-A_{c}}\right)\right]\times 100$$

where *A*_0_ and *A*_c0_ are the absorbances of the sample and the negative control, respectively, immediately after preparation, and *A* and *A*_c_ are the absorbances of the sample and the negative control after 1 h of incubation.

### 2.8 Tyrosinase Inhibition Assay

The tyrosinase inhibition assay was performed to assess the skin whitening activity of the samples because tyrosinase is the key enzyme in melanin synthesis.^2^ The samples investigated in this assay were PLGA nanocapsules with rosehip oil containing or not KDP (NC-R-KDP and NC-R, respectively) and nanocapsules without rosehip oil containing or not KDP (NC-T-KDP and NC-T, respectively) and KA solution (SOL-KA). Phosphate buffer was used as a negative control. The experiment was carried out in triplicate.

The tyrosinase inhibition assay was performed according to Zilles et al.,^11^ with modifications related to the enzyme units and the final reaction volume in the test tubes. Briefly, 500 μL of 2 mM l-tyrosine in phosphate buffer (0.1 M, pH 6.8), 400 μL of phosphate buffer (0.1 M, pH 6.8) and 50 μL of sample, diluted 3 times in phosphate buffer (such dilution was necessary regarding the absorbance reading), were added to Falcon tubes and incubated for 5 min at 37 °C. Afterward, 250 μL of phosphate buffer (0.1 M, pH 6.8) with or without the tyrosinase enzyme (0.2 mg/mL from 100 kU tyrosinase) were added to the tubes, which were then reincubated for 15 min (37 °C). The absorbance of each sample was subsequently measured with a spectrophotometer (K37-UVVIS, KASVI, Pinhais, PR, Brazil) at 405 nm to determine the dopachrome concentration. The percentage of tyrosinase inhibition was calculated via eq 4

4 %Tyrosinaseinhibition=[(A−B)÷A]×100

where *A* is the difference in absorbance between negative control samples with and without tyrosinase and where *B* is the difference in absorbance between test samples with and without tyrosinase.

### 2.9 Cell Viability and Melanin Content with Melanocytes

The cytocompatibility of the nanocapsules containing rosehip oil and KDP (NC-R-KDP) was evaluated with primary adult normal human epidermal melanocytes (NHEM) via the 3-(4,5-dimethylthiazol-2-yl)-2,5-diphenyltetrazolium bromide (MTT) assay.^33^ The cells were also used to assess melanin content.

The tissues were obtained under an IRB (Institutional Review Board) -approved Biospecimen Repository (BSR), which allows for collection of otherwise to be discarded skin. This BSR is maintained by MedStar Health Research Institute’s IRB (#2012-338). The skin samples were collected from burn patients who were undergoing autologous skin grafting. At the end of the case, if there was a small (<20 cm^2^) of autograft left, instead of being discarded, it was brought to the laboratory for processing (<1 h). The tissues were processed using a skin meshing instrument (Brennan) using a 1:1 mesh ratio and were put into 40 mL of 1X Dispase solution (Celln *T*ec) and incubated and rotated for 30 min in an Envirogenie at 39 °C. Following Dispase incubation, the epidermis was peeled from the dermis and the two were separated. The epidermis was then added to 1X trypsin (Lonza) and incubated for 15 min at 37 °C. The epidermal sheets were then neutralized with trypsin neutralizing solution (TNS) (Lonza) and then mechanically digested via pipetting up and down 50 times with a serological pipet. The suspension was then filtered through a 70 μm filter. The single cell suspension was then spun at 600 × g for 5 min to obtain a pellet. The pellet was resuspended in melanocyte media. The cells were expanded in Cnt-40 media (Cellntec) to induce rapid proliferation. During this time, fibroblasts were removed every 2–3 weeks via magnetic activated cell sorting with an MS column using human antifibroblast-specific beads (Milltenyi Biotech). Prior to assay, the cells were maintained in melanocyte media for a minimum of 2 weeks. Cells were used in assay at passage 4–7.

Normal Human Epidermal Melanocytes (NHEM), from primary culture, were maintained in fresh melanocyte medium (laboratory made) as previously described by Abdel-Malek et al.,^34^ supplemented with 1% fetal bovine serum (FBS), 0.2% amphotericin B (250 μg/mL), and a 1% penicillin (10,000 IU/mL) and streptomycin (10 mg/mL). Cells were kept at 37 °C in an incubator with 95% humidity and containing 5% CO_2_.

The cells were seeded into 96-well plates at a concentration of 1.5 × 10^4^ cells/well (NHEM) and incubated overnight at 37 °C in a humid atmosphere containing 5% CO_2_. Then, the cells were treated with nanocapsules (NC-R-KDP) at concentrations ranging from 1.5 to 50 μg/mL of KDP. The control group was treated with the respective culture medium only. After 24 h of treatment, 0.5 mg/mL MTT solution was added to each well, and the plates were incubated for 3 h at 37 °C and 5% CO_2_. The supernatant was subsequently discarded, and dimethyl sulfoxide was added to solubilize the formazan crystals. Absorbance was measured using a microplate reader at 595 and 620 nm (SpectraMax ABS, Molecular Devices). The Assays were performed in triplicate for each concentration and were repeated in three independent experiments. The results are expressed as the percentage of viable cells in relation to the control, which was calculated as follows (eq 5)­

5 Cellviability(%)=(A÷B)×100

where *A* is the mean absorbance of the samples and *B* is the mean absorbance of the control.

The melanin content in NHEM cells was quantified via a melanin content assay following a 96 h treatment with NC-R-KDP or unencapsulated KDP at concentrations of 1.5 and 3.1 μg/mL of KDP using the melanin content assay.^35^ A control group composed of culture medium only was also included in the assay. Briefly, cells were seeded in 6-well plates at a density of 2 × 10^5^ cells/well (2 mL/well) and incubated overnight at 37 °C in a humid atmosphere containing 5% CO_2_. Treatments were applied 24 h after seeding and renewed on day 3 of exposure. After 96 h of total treatment, the cells were washed twice with PBS, trypsinized, and collected. After centrifugation, the cell pellets were lysed by adding 1 N NaOH (final volume 300 μL), followed by incubation at 100 °C for 30 min to solubilize melanin. The lysates were transferred to a 96-well plate, and the absorbance was measured at 405 nm using a microplate reader (SpectraMax ABS, Molecular Devices). A standard curve was prepared using synthetic melanin diluted in 1 N NaOH. All experimental samples and standards were plated in duplicate. The melanin content in the samples was calculated using the equation obtained through the linear regression of the melanin standard curve. Assays were performed in triplicate for each concentration.

### 2.10 Cell Viability with Fibroblasts

The cytocompatibility of the nanocapsules containing rosehip oil and KDP (NC-R-KDP) was also evaluated with fibroblasts (3T3-L1) via the 3-(4,5-dimethylthiazol-2-yl)-2,5-diphenyltetrazolium bromide (MTT) assay.^33^

Fibroblasts (3T3-L1 mouse embryonic fibroblast cells) were maintained in Dulbecco’s modified Eagle medium (DMEM) supplemented with 10% fetal bovine serum (FBS), 0.2% amphotericin B 250 μg/mL, and a 1% solution of penicillin (10,000 IU/mL) and streptomycin (10 mg/mL). The cells were kept at 37 °C in an incubator with 95% humidity and containing 5% CO_2_.

The cells were seeded into 96-well plates at a concentration of 2 × 10^4^ cells/well and incubated overnight at 37 °C in a humid atmosphere containing 5% CO_2_. Then, the cells were treated with nanocapsules (NC-R-KDP) at concentrations ranging from 1.5 to 50 μg/mL of KDP. The control group was treated with the corresponding culture medium only (DMEM for 3T3). After 24 h of treatment, 0.5 mg/mL MTT solution was added to each well, and the plates were incubated for 3 h at 37 °C and 5% CO_2_. The supernatant was subsequently discarded, and dimethyl sulfoxide was added to solubilize the formazan crystals. The absorbance was measured using a microplate reader at 570 and 630 nm (SpectraMax M5, Molecular Devices) (3T3 cells). Even though the MTT protocol was the same as the one used for melanocytes, the assays were read at slightly different wavelength pairs because of different laboratories protocols; comparisons were performed within each cell type’s assay, not across the two cell systems. Assays were performed in triplicate for each concentration and repeated in three independent experiments. The results are expressed as the percentage of viable cells in relation to the control and were calculated via eq 5, Section 2.9.

### 2.11 Statistical Analysis

The nanocapsules were developed in triplicate of batches, and the experiments were carried out in triplicate unless otherwise stated. The results are expressed as mean ± standard deviation. Differences between groups were evaluated by one-way analysis of variance (ANOVA) followed by a posthoc test for multiple comparisons (Tukey’s test) or by two-way ANOVA with Tukey’s test for comparisons across multiple time points. For comparisons between two groups, Student’s *t*-test was applied. GraphPad Prism version 8.0.2 (San Diego, CA, USA) was used to conduct the analysis. A p value ≤0.05 was considered statistically significant.

## 3 Results and Discussion

### 3.1 HPLC-UV Methods Validation

The HPLC-UV methods were specific for KDP and KA quantification, without interference of other constituents of the formulations, such as rosehip oil, polysorbate 80 and the different polymers tested.

The method for KDP quantification showed linearity between 1 and 30 μg/mL, with an *R* value of 0.9997. The intraday precision (repeatability) and intermediate precision results were considered adequate, with standard deviations of less than 5%, in accordance with the International Conference on Harmonization (ICH) guidelines Q2 (R1).^28^ The accuracy assay led to a mean recovery of 98.73% ± 1.47%.

The method for KA quantification showed linearity between 1 and 50 μg/mL, with an *R* value of 0.9997. The intraday precision (repeatability) and intermediate precision results were considered adequate, with standard deviations of less than 5% in accordance with the International Conference on Harmonization (ICH) guidelines Q2 (R1).^28^ The accuracy assay led to a mean recovery of 100.85% ± 2.12%. The KA quantification limit was 0.7789 μg/mL.

### 3.2 Nanocapsules Development and Characterization

The developed nanocapsules resulted in homogeneous milky white formulations with bluish brightness and no precipitates, regardless of the polymer used. The employed method was the interfacial deposition of the preformed polymer, which is a bottom-up technique.^14^ This technique has advantages due to its simplicity, reproducibility and high drug loading capacity.^18^ Table 2 shows the average droplet size and size distribution, zeta potential and pH of the three batches of each formulation immediately after obtainment, as well as after storage.

**Table 2:** Characterization of Kojic Acid Dipalmitate and Rosehip Oil-Loaded Nanocapsules Obtained with Eudragit RS100 (NC ERS100), Eudragit S100 (NC ES100), poly­(ε-Caprolactone) (NC PCL) and poly­(d,l-lactide-*co*-glycolide)^a^

		NC ERS100	NC ES100	NC PCL	NC PLGA
day 0	Z Ave (nm)	146 ± 1	198 ± 5	194 ± 1	193 ± 2
	PDI	0.150 ± 0.017	0.144 ± 0.023	0.089 ± 0.004	0.148 ± 0.029
	ZP (mV)	+8.87 ± 0.83	–6.00 ± 1.03	–3.87 ± 0.29	–7.37 ± 0.36
	pH	4.43 ± 0.39	2.79 ± 0.03	3.63 ± 0.09	2.55 ± 0.03
room temperature storage (25 °C)					
day 30	Z Ave (nm)	142 ± 5	370 ± 133	196 ± 1	194 ± 5
	PDI	0.154 ± 0.032	0.252 ± 0.035	0.115 ± 0.023	0.151 ± 0.007
	ZP (mV)	+12.37 ± 1.55	–11.21 ± 1.29	–8.52 ± 3.06	–11.26 ± 1.58
	pH	3.73 ± 0.48*	3.21 ± 0.08	3.37 ± 0.38	2.81 ± 0.12
day 90	Z Ave (nm)	138 ± 1	4249 ± 1312*	200 ± 5	199 ± 6
	PDI	0.143 ± 0.003	0.766 ± 0.401*	0.110 ± 0.009	0.224 ± 0.030
	ZP (mV)	+17.03 ± 9.20*	–14.97 ± 0.29*	–8.99 ± 4.21	–13.10 ± 0.82
	pH	4.92 ± 0.04	2.74 ± 0.08	3.07 ± 0.06*	3.46 ± 0.06*
day 180	Z Ave (nm)	143 ± 4	8519 ± 347*	202 ± 2	215 ± 6
	PDI	0.163 ± 0.019	0.634 ± 0.202*	0.145 ± 0.095	0.207 ± 0.037
	ZP (mV)	+14.15 ± 4.67	–22.13 ± 4.92*	–13.8 ± 1.15*	–15.10 ± 1.82*
	pH	4.07 ± 1.10	3.10 ± 0.02	4.28 ± 0.57	3.49 ± 0.15*
refrigerated storage (4 °C)					
day 30	Z Ave (nm)	142 ± 1	194 ± 7	200 ± 4	200 ± 7
	PDI	0.144 ± 0.025	0.155 ± 0.009	0.084 ± 0.018	0.187 ± 0.026
	ZP (mV)	+9.32 ± 2.84	–11.13 ± 1.31	–8.84 ± 2.10	–9.68 ± 0.33
	pH	3.61 ± 0.16*	2.85 ± 0.06	3.64 ± 0.37	2.61 ± 0.03
day 90	Z Ave (nm)	144 ± 3	196 ± 5	206 ± 6	210 ± 7
	PDI	0.162 ± 0.014	0.159 ± 0.017	0.117 ± 0.005	0.195 ± 0.008
	ZP (mV)	13.83 ± 9.26	–11.13 ± 0.60	–8.37 ± 1.11	–8.23 ± 0.02
	pH	5.07 ± 0.49	2.95 ± 0.14	3.54 ± 0.02	2.93 ± 0.10
day 180	Z Ave (nm)	143 ± 4	229 ± 53	205 ± 6	216 ± 12
	PDI	0.149 ± 0.021	0.158 ± 0.015	0.123 ± 0.018	0.192 ± 0.037
	ZP (mV)	9.85 ± 3.04	–13.87 ± 0.76*	–9.21 ± 2.46	–9.88 ± 1.62
	pH	3.98 ± 0.29	2.98 ± 0.13	4.47 ± 0.67*	2.93 ± 0.09

a (NC PLGA), immediately after obtainment and after 30, 90, and 180 days of storage at 4 °C and 25 °C. Represents a significant difference from day 0 (*p* ≤ 0.05).

The laser diffraction technique measures the size of a particle by scattering light in a similar way to that of the particles under investigation, and it is a suitable technique to measure particles with diameters larger than 1 μm.^36^ Therefore, our formulations were first investigated through this technique to verify the absence of micrometric particles. All formulations presented a monomodal nanometric size distribution, as shown in Figure 1, and were further analyzed by the dynamic light scattering technique, as in this technique, particles must have diameters smaller than 1 μm.^36^ Dynamic light scattering measures the size of a particle by determining its hydrodynamic radius by scattering light with the same intensity as the particles under investigation while in dispersion; it is an adequate analysis for nanometric particles.^36^ Polymeric nanocapsules are reported to have average diameter ranging from 70 to 300 nm.^37^ Indeed, the developed polymeric nanocapsules presented particle size smaller than 200 nm regardless of the polymer used, with a monomodal distribution and PDI values considered adequate. The size values found in the present investigation are similar to those reported in a previous study by Contri and co-workers, who developed nanocapsules with Eudragit RS100 and rosehip oil.^12^

**Figure 1:**
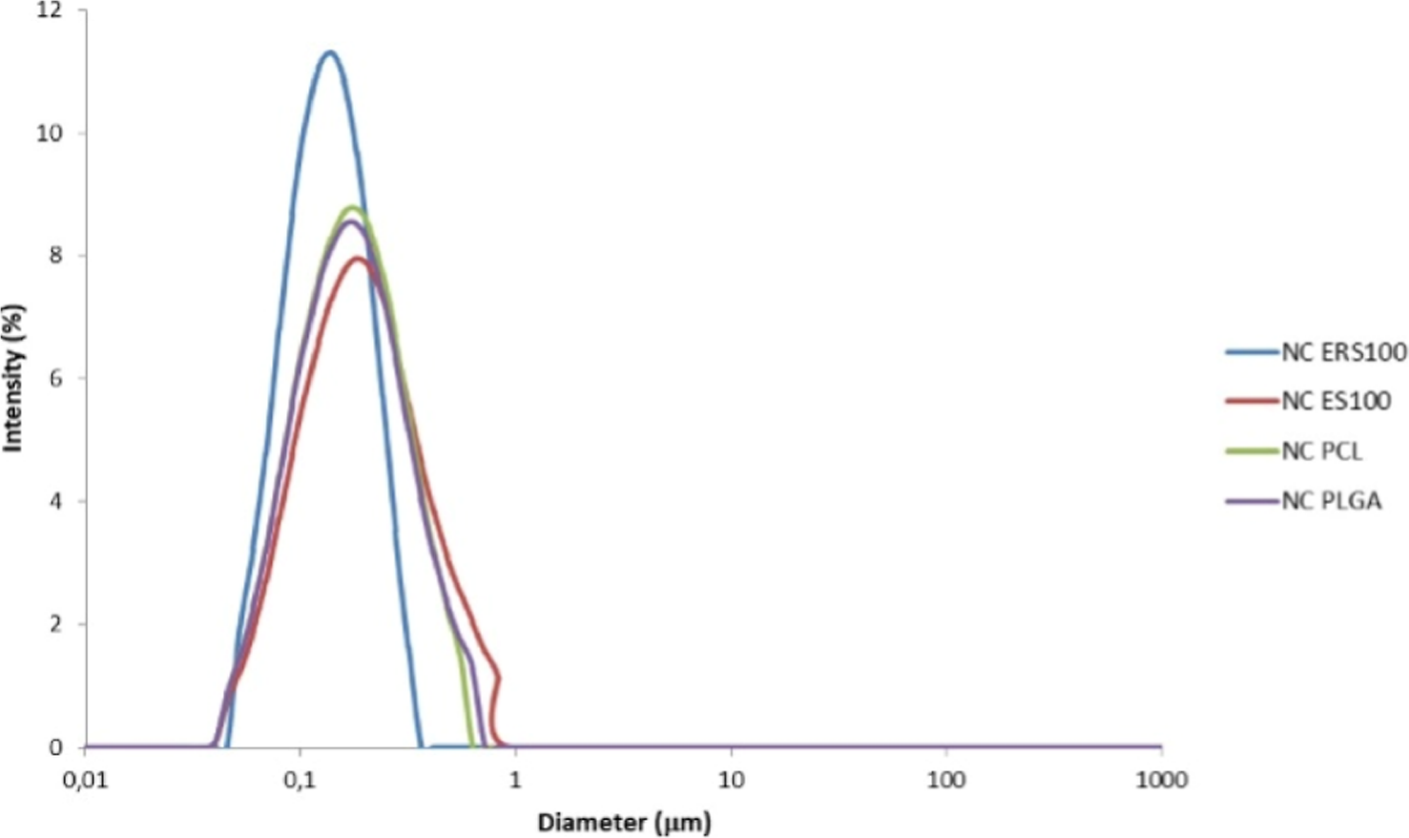
Particle size distribution (laser diffraction technique) of KDP and rose-hip oil-loaded nanocapsules obtained with Eudragit RS100 (NC ERS100), Eudragit S100 (NC ES100), poly­(ε-caprolactone) (NC PCL) and poly­(d,l-lactide-*co*-glycolide) (NC PLGA).

The zeta potential of a formulation indicates its surface charge and estimates the kinetic stability of formulations.^38^ The NC ERS100 showed a positive zeta potential, which was expected since it was formulated with a cationic polymer,^12,39^ whereas all the others (NC ES100, NC PCL and NC PLGA) were formulated with anionic polymers and presented negative values.^14–16,40^ Since all the formulations presented on day zero zeta potential values lower than 10 mV in module, the stability of the system was attributed mainly to steric effects.^38^ Regarding the pH values of the nanocapsules, it varied from 2.55 ± 0.03 to 4.43 ± 0.39. Since the formulations are intended for topical use and the skin surface pH is approximately 5.5, pH values between 4 and 7 are ideal for topical formulations.^41^ Although maintaining an acidic pH is considered essential for topic formulations, as it helps preserving the skin’s acid mantle, optimal pH ranges and full implications for skin physiology are yet not completely understood.^42^ Acidic pH as low as 2.5 could cause skin irritation, but Hwang et al. showed that irritation relies not only on pH, but also on the acid concentration of the formulation.^43^ On the other hand, there are also reports about low pH values (around 3) promoting cellular turnover and helping the improvement of the skin’s barrier function.^44^ It is important to mention that although the pH values of the nanocapsules suspensions were acidic, pH adjustments can be performed after their incorporation into cosmetic vehicles.

The KDP content of NC ERS100, NC ES100, NC PCL and NC PLGA was approximately 90%, indicating that approximately 1 mg (for a total of 10 mg) of the active substance was degraded or not recovered during manufacturing. The encapsulation efficiency was above 99% regardless of the polymer used, suggesting the high affinity of KDP with rosehip oil^11^ and also suggesting the suitability of the nanocapsules to encapsulate KDP.

In summary, it was possible to develop KDP and rosehip oil nanocapsules with adequate features for all the tested polymers. The selection of the polymers to develop the nanocapsules was based on their biocompatibility and possibility to form a barrier against the external aqueous media of the suspensions, considering the tendency of KDP to undergo hydrolysis. Eudragit polymers are used to develop biocompatible and stable nanoparticles.^16^ Eudragit RS100 is a cationic polymer, which may improve the formulation adhesion with the negative charge of the skin tissue,^16,40^ whereas Eudragit S100 is an anionic and pH-dependent polymer, which can have a targeted release.^16^ Poly­(ε-caprolactone) is also a biocompatible polymer, and it is a semicrystalline polymer; because of that, its degradation can be delayed, resulting in more stable formulations,^18^ and its molecular weight is directly related to its crystallinity.^15^ PLGA is a biocompatible and nontoxic synthetic polymer that can be used to control the release of active substances.^15^ The nanocapsules stability (protection from KDP hydrolysis and maintenance of nanometric features) was further analyzed to select the most promising formulation for the activity assays.

### 3.3 Nanocapsules Stability

The four different formulations were stored in amber glass flasks at room temperature and at refrigerated storage, and they were recharacterized after 30, 90, and 180 days. Regarding the formulations aspect, no changes were observed throughout the 180 days, as they remained with homogeneous milky white aspect, with bluish brightness, regardless of the polymer used. Table 2 shows the analyzed parameters of particle size and size distribution, zeta potential and pH. The particle size and size distribution of the formulations developed with Eudragit RS100, PCL and PLGA did not show significant differences (*p* ≥ 0.05) compared to day 0, neither at room temperature storage nor at refrigerated storage. However, the formulation developed with Eudragit S100 was not stable at room temperature storage, and an increase in its particle size and size distribution was observed, suggesting aggregation of the particles. Unlike the other polymers tested, Eudragit S100 is a pH-sensitive polymer, soluble at pH 7.^16^ In an acidic environment, such as the one in the nanocapsules formulation, the polymer will be insoluble, weakly ionized, and will exhibit low molecular mobility, which could have influenced in the formulation’ stability, especially at room temperature. The pH values of the formulations were found to be in the range of 2.61 ± 0.03 to 5.07 ± 0.49, which was similar to the values found on day 0. Some slight changes were also observed for the zeta potential, especially at room temperature storage for 90 days or more.

Drug content of the formulations throughout time and under both storage conditions is depicted in Figure 2. The refrigerated storage promoted the stability of the active substance content for at least 30 days for all the formulations. However, at 180 days of storage, only the formulation composed of PLGA maintained its initial KDP content. At room temperature, the PLGA formulation also stood out. Even though after 30 days of storage all of the formulations showed significant decay in KDP content in comparison to day 0, the one with PLGA (NC PLGA) was found to contain around 80% of the initial KDP content, which is higher than a prior developed nanoemulsion containing KDP and rosehip oil (around 70% of the initial content).^11^ The KDP is the dipalmitic ester of kojic acid, and hydrolysis reaction can occur during storage, leading to the formation of kojic acid monopalmitate and kojic acid. The hydrolysis reaction involves the cleavage of labile bonds when in contact with water, such as the case of esters in water.^45^ Since heat can accelerate this reaction,^45^ it can explain why the nanocapsules stored at refrigerated temperature were able to retain their original KDP content. Another possibility for losses of KDP content at room temperature storage could be the oxidation of the molecule through a ring opening mechanism, which can occur in liquid oxidative stress conditions.^24^

**Figure 2:**
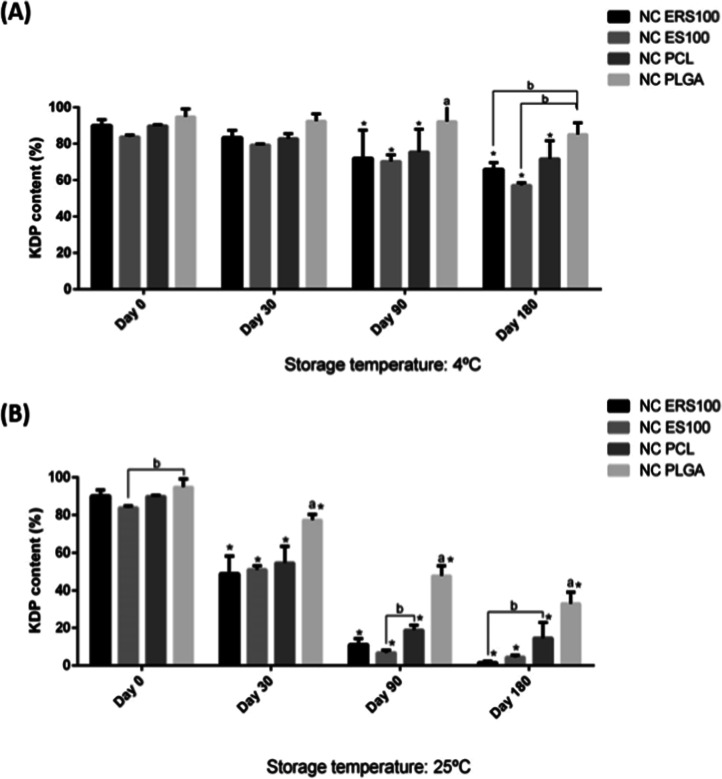
Kojic acid dipalmitate (KDP) content (%) in KDP and rose-hip oil-loaded nanocapsules obtained with Eudragit RS100 (NC ERS100), Eudragit S100 (NC ES100), poly­(ε-caprolactone) (NC PCL) and poly­(d,l-lactide-*co*-glycolide) (NC PLGA) after storage at refrigerated storage (A); and at room temperature storage (B). * represents a significant difference from day 0 (*p* ≤ 0.05). “a” indicates a significant difference from all the other formulations for a specific period of analysis (*p* ≤ 0.05). “b” indicates a significant difference between formulations (*p* ≤ 0.05).

It is important to mention that decays in KDP content do not necessarily indicate losses in activity since it can be hydrolyzed into kojic acid.^11^ For this reason, the kojic acid (KA) content of the formulations stored at room temperature was analyzed throughout time. These results corroborate our hypothesis that KDP indeed undergoes hydrolysis and becomes kojic acid, as a rise in the KA content was observed with a decrease in the KDP content, as shown in Figure 3. Moreover, NC PLGA was the one that led to less formation of KA at room temperature, highlighting the use of PLGA as a polymer to develop high-stability KDP nanocapsules. To the best of our knowledge, this is the first study investigating KDP degradation into KA.

**Figure 3:**
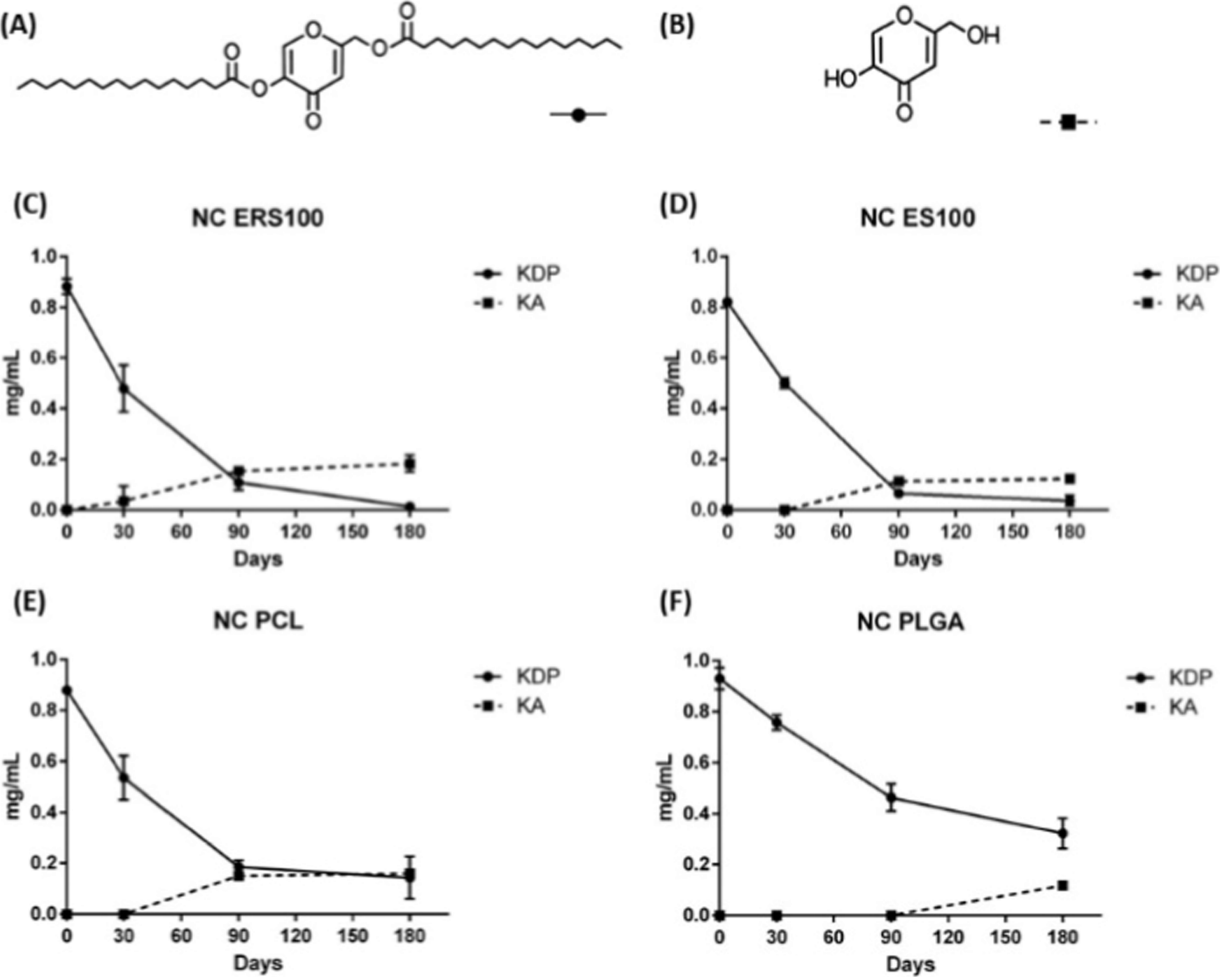
Molecules of kojic acid dipalmitate (KDP) (A) and kojic acid (KA) (B). KDP and KA content (mg/mL) in (C) Eudragit RS100 nanocapsules (NC ERS100), (D) Eudragit S100 nanocapsules (NC ES100), (E) poly­(ε-caprolactone) nanocapsules (NC PCL) and (F) poly­(d,l-lactide-*co*-glycolide) nanocapsules (NC PLGA) stored at 25 °C for up to 180 days.

The KDP crystal content of the formulations was also investigated at room temperature in immobilized samples of the four nanocapsules formulations, since KDP is a highly lipophilic molecule and the KDP nanocapsules are in aqueous media. Lipophilic substances can agglomerate and crystallize if they are not properly soluble. Lipophilic substances can leak from the particles during storage, hindering the total drug content in the stored nanocapsules suspension.^25^ The maximum value of KDP crystals observed after 180 days of storage was 2.75 ± 3.01% (NC PLGA), but it was not significantly different from that of the other formulations (NC ERS100 0.0 ± 0.0%, NC ES100 0.01 ± 0.02%, NC PCL 0.0 ± 0.0%). The results obtained for the PLGA nanocapsule were lower than those reported for a 1 mg/mL KDP and rosehip oil nanoemulsion, which formed approximately 10% of KDP crystals after 30 days of storage at room temperature.^11^ Therefore, the nanocapsules showed less crystal formation in a longer period of study, suggesting greater stability.

Since the properties of the formulations did not differ during stability (with the exception of the NC ES100), the choice of the best formulation was made regarding the KDP content throughout time. The PLGA nanocapsule was the formulation that showed the most promising stability results regarding KDP content. Indeed, all the polymers tested in this study are biocompatible and have been reported to produce stable formulations, but in the present investigation, PLGA stood out. PLGA nanocapsules containing rose hip oil and KDP (now named NC-R-KDP) were selected for further studies of preliminary efficacy and safety.

PLGA is a biocompatible and nontoxic synthetic polymer that can be used to control the release of active substances.^15^ One of the advantages of PLGA is that in physiological systems, it can suffer hydrolysis, resulting in the formation of metabolites that are easily metabolized,^15^ indicating the safety of this polymer for human use. Moreover, it has adequate biodegradability and controlled delivery properties and can easily encapsulate lipophilic compounds.^15^ PLGA nanoparticles have already been described for cosmeceutical applications, such as an antiaging formulation with glycyrrhizic acid,^46^ a provitamin C formulation with antiaging and skin whitening properties,^47^ and as drug-nanocarriers formulation for quercetin, which can attenuate UVB damage in the skin.^48^

### 3.4 In Vitro Skin Permeation Assay

The nanocapsules developed were intended for topical application. Hence, their influence on the skin permeation profile of KDP was evaluated. The porcine membrane was used in this assay due to its anatomical characteristics and permeability similarities with human skin.^49^ The receptor medium was composed of 7.5% polysorbate 80 in water, based on a previous study on KDP skin permeation.^11^ Prior to the assay, KDP solubility in the receptor medium was assessed and a value of 230 μg/mL was obtained, demonstrating that the receptor medium can solubilize the entire KDP amount if 100% of the applied dose permeates. Moreover, it is important to mention that the experiment was performed under infinite dose regimen, and therefore skin saturation is expected.^50,51^

Figure 4 shows the amount of KDP from 1 mg/mL nanocapsules (NC-R-KDP) that permeated through the skin after 12 and 24 h of contact with the porcine membrane, as well as the amount of KDP retained on the stratum corneum, epidermis and dermis. The nanocapsules formulation was compared with a 1 mg/mL KDP dispersion (D-KDP), where KDP was found to be unencapsulated, to verify whether it would lead to differences in skin permeation.

**Figure 4:**
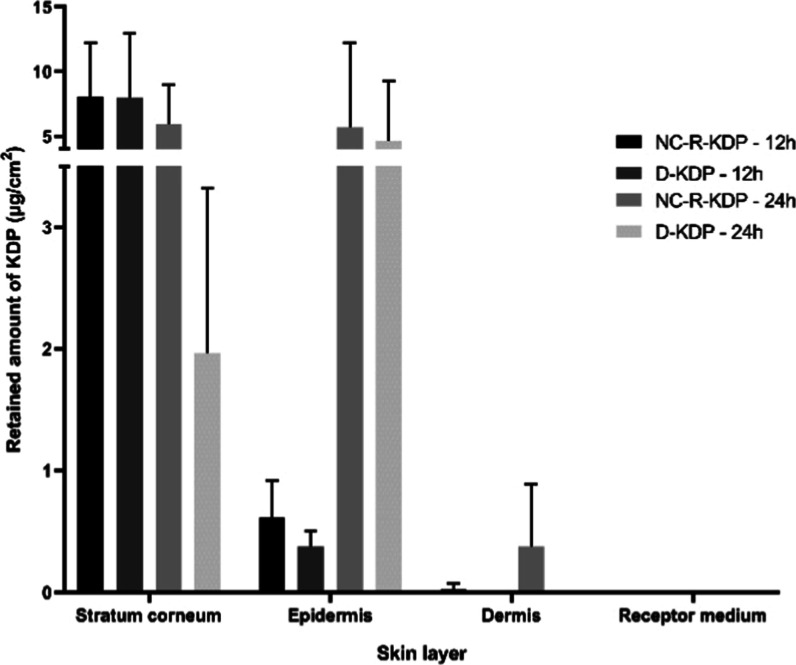
Kojic acid dipalmitate (KDP) concentrations in the receptor medium, dermis, epidermis and stratum corneum after 12 h and 24 h treatment with KDP and rosehip oil-loaded nanocapsules (NC-R-KDP) and KDP dispersion (D-KDP).

After the excess formulation was removed, the stratum corneum was removed, the epidermis and dermis were separated, and the KDP retained in each of the skin layers was quantified. Results showed that there were no significant differences (*p* ≥ 0.05) between the quantified KDP from formulations NC-R-KDP and D-KDP in the different skin layers, regardless of the treatment duration, indicating that the nanocapsules did not hinder KDP skin permeation. It is important to notice that the nanocapsules led to higher mean values of KDP in the viable layers (viable epidermis and dermis). Regarding the epidermis, 0.61 μg/cm^2^ and 5.70 μg/cm^2^ of KDP was retained after 12 and 24 h, respectively, for NC-R-KDP, and 0.38 μg/cm^2^ and 4.63 μg/cm^2^ after 12 and 24 h, respectively, for D-KDP. In the dermis, 0.03 μg/cm^2^ and 0.38 μg/cm^2^ of KDP was retained after treatment with NC-R-KDP after 12 and 24 h, respectively, whereas it was not possible to quantify the active substance after treatment with D-KDP.

The intended target of the formulations is the basal layer of the epidermis, where melanocytes are located.^4^ Melanin is produced by the melanosomes inside the melanocyte cells.^2,4^ Therefore, it was observed that nanocapsules formulation (NC-R-KDP) was able to overcome the main barrier of the skin, the stratum corneum, allowing KDP to reach the epidermis. Moreover, it was possible to quantify KDP, when applied in the form of nanocapsules, in the dermis, suggesting that the active substance permeated the epidermis and hence reached its basal layer. Extending the treatment from 12 to 24 h did not result in statistically significant changes in KDP retention for either NC-R-KDP or D-KDP (*p* > 0.05). However, both formulations showed a trend toward higher KDP retention in viable layers. This result indicates a time-dependent diffusion of KDP, without systemic permeation. It is important to notice that KDP from the tested formulations did not permeate the full thickness of the skin, as it was not possible to quantify it in the receptor medium, indicating that it did not go down to the subcutaneous fat. Moreover, the low quantity of KDP in the dermis indicates a low probability of KDP reaching the bloodstream and therefore has a low probability of causing systemic effects. These findings suggest safety of the formulations.

When a rosehip oil and KDP nanoemulsion (1 mg/mL KDP) was evaluated for skin permeation with 12 h treatment, KDP was shown to be retained in the epidermis at approximately 1.2 μg/cm^2^.^11^ This result is slightly higher than that reported in the present study. Different nanostructures can lead to different outcomes. While nanocapsules can better protect the active substance encapsulated and control its release, nanoemulsions can have greater affinity with the skin because of their lipophilic characteristics, which could result in higher permeation rates.^37,52^ The nanoemulsion led to KDP retention in the stratum corneum at approximately 2.5 μg/cm^2^,^11^ while the nanocapsules (NC-R-KDP) developed in the present study showed a KDP retention of 8 μg/cm^2^ ± 4, suggesting that the polymeric nanocapsules could be forming a KDP deposit in the stratum corneum, which could lead to a controlled release and prolonged action.^52^

### 3.5 Antioxidant Activity

The antioxidant activity of the PLGA nanocapsules was investigated by two different assays: the DPPH assay and the β-carotene/Linoleic acid assay. These two assays investigate the antioxidant activity through different mechanisms. While in the first one the reducing capacity of the formulations can be observed, with the reduction of the DPPH radical (radical scavenging activity) being correlated with antioxidant activity,^31^ in the latter the protective capacity of a formulation to avoid the oxidation of the β-carotene/Linoleic acid system is investigated.^53^ The activity of the PLGA nanocapsules containing 1 mg/mL KDP and rosehip oil (NC-R-KDP) was compared to both the activity of the nanocapsules containing each of the active ingredients alone (NC-R and NC-T-KDP, for rosehip oil and KDP, respectively), and the activity of the completely unloaded nanocapsules, without the active substances (NC-T). It was also compared to a 0.23 mg/mL KA solution (SOL-KA), which corresponds to 1 mg/mL of KDP.

The KDP molecule consists of two palmitic acid molecules attached to the two hydroxyl groups of kojic acid,^3^ hence, 1 mol of KDP generates 1 mol of kojic acid. KDP has a molecular weight of 618.9 g/mol, while kojic acid has a molecular weight of 142.11 g/mol.^3^ Therefore, a 0.23 mg/mL solution of kojic acid is expected to have similar activity as a 1 mg/mL KDP formulation.

The DPPH assay investigates the antioxidant capacity of a formulation or substance through its capacity to reduce the DPPH radical.^31^ Our results are depicted in Figure 5A. The NC-R-KDP and NC-R samples presented the highest antioxidant activity, with approximately 20% of inhibition when used at 25 μg/mL, whereas all the other samples presented less than 5% of inhibition at the same concentration. Therefore, the encapsulation increased the antioxidant activity of KDP. In agreement with this finding, a KDP nanoemulsion showed greater antioxidant activity compared to a KDP dispersion,^11^ and also, a kojic acid nanostructured lipid carriers presented greater antioxidant activity compared to unencapsulated kojic acid.^54^

**Figure 5:**
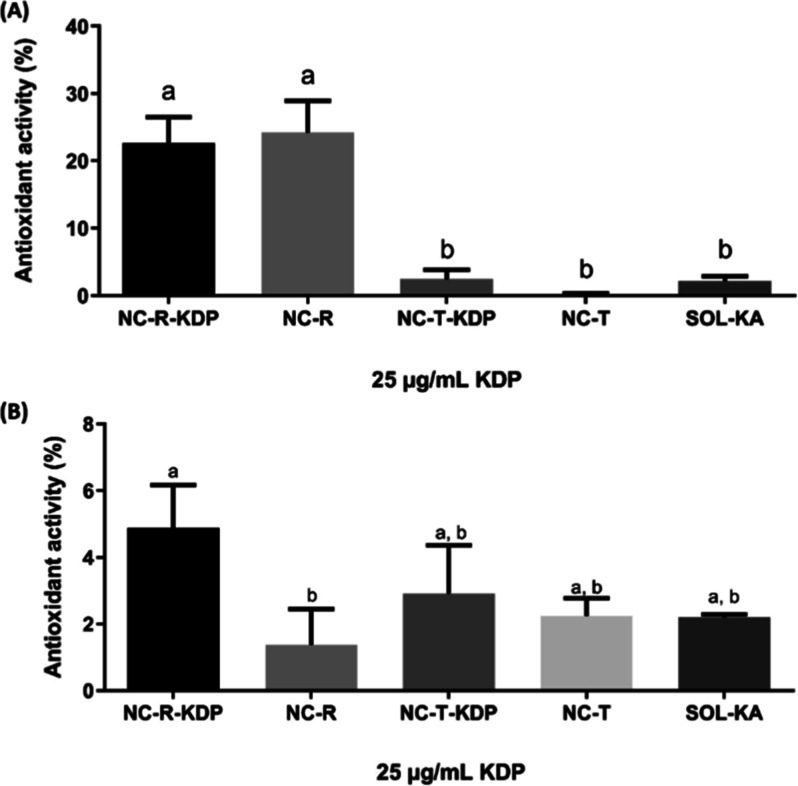
Antioxidant activity percentage of nanocapsules containing KDP and rosehip oil (NC-R-KDP), nanocapsules containing each of the active ingredients alone (NC-R and NC-T-KDP, for rosehip oil and KDP, respectively), nanocapsules without the active substances (NC-T), and KA solution (SOL-KA, 0.23 mg/mL) in the DPPH assay (A) and in the β-carotene/linoleic acid assay (B). For SOL-KA, the sample was prepared to match the KDP-equivalent concentration of NC-R-KDP. Formulations without KDP (NC-R and NC-T) were tested in equal volumes to their KDP-containing counterparts. Different letters indicate significant difference (*p* ≤ 0.05).

Comparing the 1 mg/mL KDP nanocapsules with and without rosehip oil, NC-R-KDP and NC-T-KDP in the DPPH assay, the first one had significantly higher activity, approximately 8 times higher. Caprylic/capric triglycerides are composed of medium-chain triglycerides, such as unsaturated fatty acids. Unsaturated fatty acids can have antioxidant activity.^55^ Comparing both caprylic/capric triglyceride nanocapsules, NC-T-KDP (containing KDP) and NC-T (not containing KDP), although there were no significant differences between them, the one with KDP had higher activity. It is important to notice that the formulations with caprylic/capric triglycerides had antioxidant activity similar to that of the KA solution (SOL-KA), which suggests that KDP does have activity, but such activity is not necessarily increased when in combination with rosehip oil or caprylic/capric triglycerides. It is also relevant to mention that the SOL-KA was tested at 0.23 mg/mL, corresponding to 1 mg/mL of KDP.

The two formulations with the highest activity in the DPPH assay were those containing rosehip oil, which indicates that the oil is indeed related to such activity, in accordance with the findings of our previous study.^11^ Rosehips are reported to have a rich composition full of substances with antioxidant activity, showing potential for use in skin products and preventing skin aging.^8,56^ The nanoencapsulation of rosehip oil can protect it from oxidation, and it has been previously described, showing that it can prevent losses in activity.^12^

The β-carotene/linoleic acid assay is considered an adequate assay for the determination of antioxidant activity from lipophilic substances,^57^ as is the case of KDP. The results obtained in this assay are presented in Figure 5B, and the samples were tested to reach a final concentration equivalent to 25 μg/mL of KDP.

Unlike what was observed in the DPPH assay, in the β-carotene/linoleic acid assay, the antioxidant activity of the samples was attributed mainly to KDP instead of rosehip oil. The differences in the results are probably related to the fact that the techniques involve different mechanisms of antioxidant activity.^53,57^ The KDP and rosehip oil nanocapsules (NC-R-KDP) showed an antioxidant activity of 4.88% ± 1.29, whereas the same nanocapsules without KDP (NC-R) presented an activity of 1.38% ± 1.07. Although no significant difference was observed between NC-R-KDP and NC-T-KDP (*p* ≥ 0.05), NC-R-KDP showed a tendency to have greater antioxidant activity than NC-T-KDP, which suggests benefits from the coencapsulation of both KDP and rosehip oil over the sole encapsulation of KDP. This result is in accordance with the results obtained in the DPPH assay, which also suggests benefits of the coencapsulation.

Moreover, when the nanocapsules with KDP and rosehip oil (NC-R-KDP) were compared with the KA solution, in both the DPPH and the β-carotene/Linoleic acid assays, the nanocapsule led to greater antioxidant activity than the kojic acid solution (SOL-KA), which was used at a concentration corresponding to the KDP concentration used. Such findings demonstrate that nanoencapsulated KDP stands out for its antioxidant activity. It was possible to coencapsulate two active antioxidant substances that act through different pathways, highlighting the potential of the developed nanocapsules suspension.

### 3.6 Tyrosinase Inhibition Assay

Tyrosinase is the key enzyme in melanin synthesis,^2^ catalyzing the first two steps of melanin formation: the hydroxylation of tyrosine in dihydroxyphenylalanine (DOPA) and the oxidation of DOPA into dopaquinone, which is then converted into dopachrome and eventually into melanin.^2,4^ The tyrosinase inhibition assay quantifies the formed dopachrome under UV light at 405 nm;^11,58^ hence, it is a specific assay to investigate skin whitening and/or lightning agents on the basis of dopachrome formation.

The tyrosinase inhibition assay results are shown in Figure 6. The effects of 1 mg/mL KDP nanocapsules (NC-R-KDP) or the other samples (NC-R, NC-T-KDP, NC-T and SOL-KA) were investigated at a final concentration of 13.75 μg/mL of KDP. The polymeric nanocapsules containing KDP inhibited tyrosinase enzyme in 17.01% ± 1.51% and 20.07% ± 1.49% for NC-R-KDP and NC-T-KDP, respectively. This result correlates with the findings of Zilles et al. (2023), who tested a nanoemulsion containing KDP and rosehip oil that presented approximately 20% of tyrosinase inhibition.^11^ However, the concentration of the polymeric nanocapsules used in the present study was approximately 12 times lower than that of the KDP nanoemulsion tested in the study of Zilles et al. (2023), highlighting the great potential of the developed PLGA nanocarrier.

**Figure 6:**
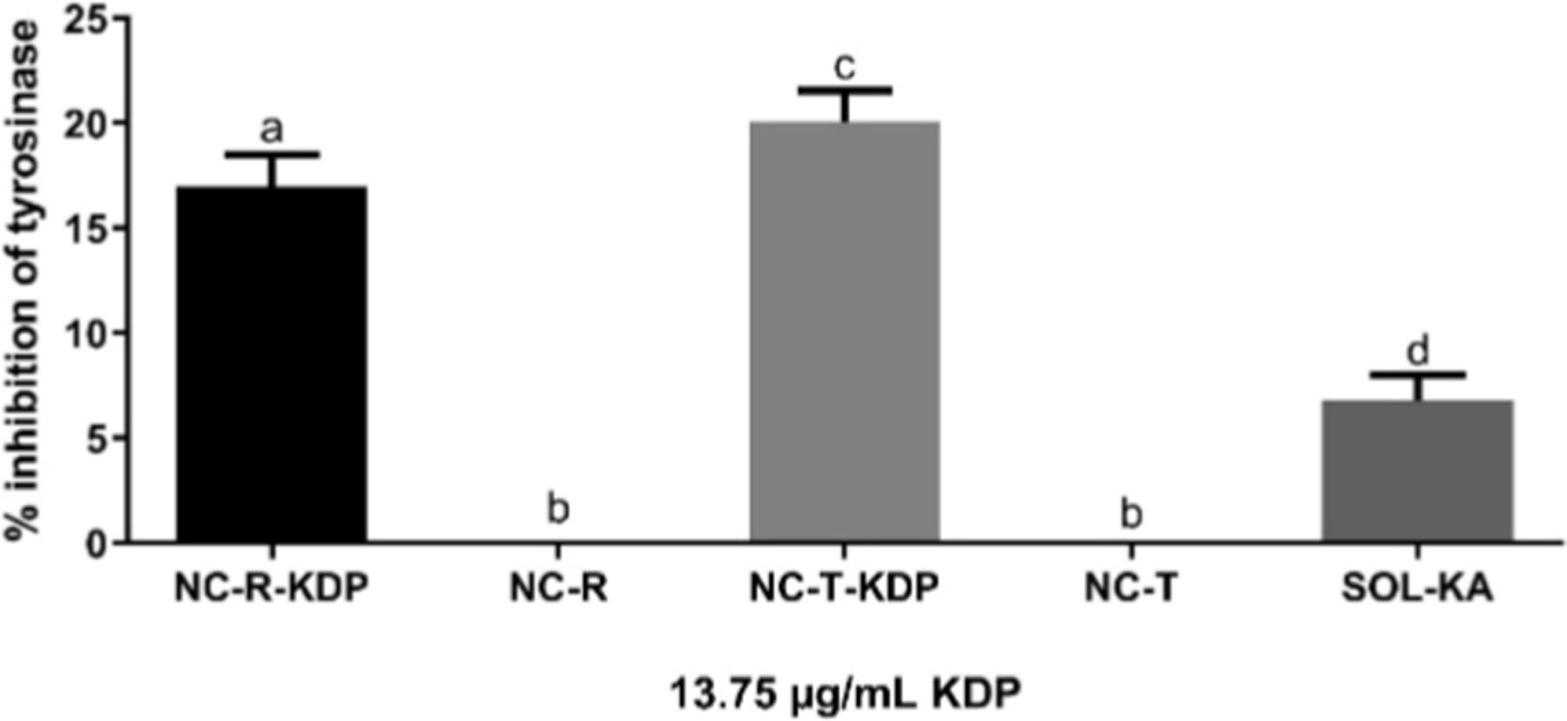
Tyrosinase inhibition percentage of nanocapsules containing KDP and rosehip oil (NC-R-KDP), nanocapsules containing each of the active ingredients alone (NC-R and NC-T-KDP, for rosehip oil and KDP, respectively), nanocapsules without the active substances (NC-T), and KA solution (SOL-KA, 0.23 mg/mL). For SOL-KA, the sample was prepared to match the KDP-equivalent concentration of NC-R-KDP. Formulations without KDP (NC-R and NC-T) were tested in equal volumes to their KDP-containing counterparts. Different letters indicate significant difference (*p* ≤ 0.05).

When comparing NC-R-KDP with SOL-KA at 13.75 μg/mL, the nanocapsules were also significantly (*p* ≤ 0.01) more efficient than SOL KA (6.78% ± 1.24), which could be explained by the smaller size of the particles and higher contact surface, enabling more contact with the enzyme.^37,52^ Khezri and co-workers (2020) studied kojic acid solid lipid nanoparticles and also compared them to a KA solution.^59^ The nanoparticles exhibited an IC50 of 3.84 ± 0.122 μg/mL for tyrosinase inhibition, whereas the KA solution had an IC50 of 18.31 ± 9.37 μg/mL.^59^ This result shows a more potent effect for the nanoformulation in comparison to the free drug, similar to what we report in the present study. Finally, the KDP unloaded nanocapsules (NC-R and NC-T) showed no tyrosinase inhibitory activity, in agreement with the findings of a rosehip oil nanoemulsion.^11^ Considering the composition of rosehip oil, which is rich in antioxidant substances such as ascorbic acid and phenolic compounds^8,12^ and that antioxidant activity is one of the multiple depigmenting mechanisms,^5^ the skin whitening mechanism of rosehip oil is possibly not related to direct inhibition of tyrosinase, since NC-R did not show tyrosinase inhibition. Additionally, it should be considered that the oil is completely surrounded by a polymeric wall, which could interfere with the interaction between the oil and the enzyme.

### 3.7 Cell Viability Assay

The cell viability of fibroblasts (3T3-L1 mouse embryonic fibroblasts) and melanocytes (NHEM) was investigated after a 24 h treatment with the NC-R-KDP formulation. The nanocapsules were tested at concentrations ranging from 1.5 μg/mL to 50 μg/mL of KDP. The results are displayed in Figure 7. NC-R-KDP showed cytocompatibility at concentrations of up to 3.1 μg/mL of KDP in both fibroblasts and melanocytes.

**Figure 7:**
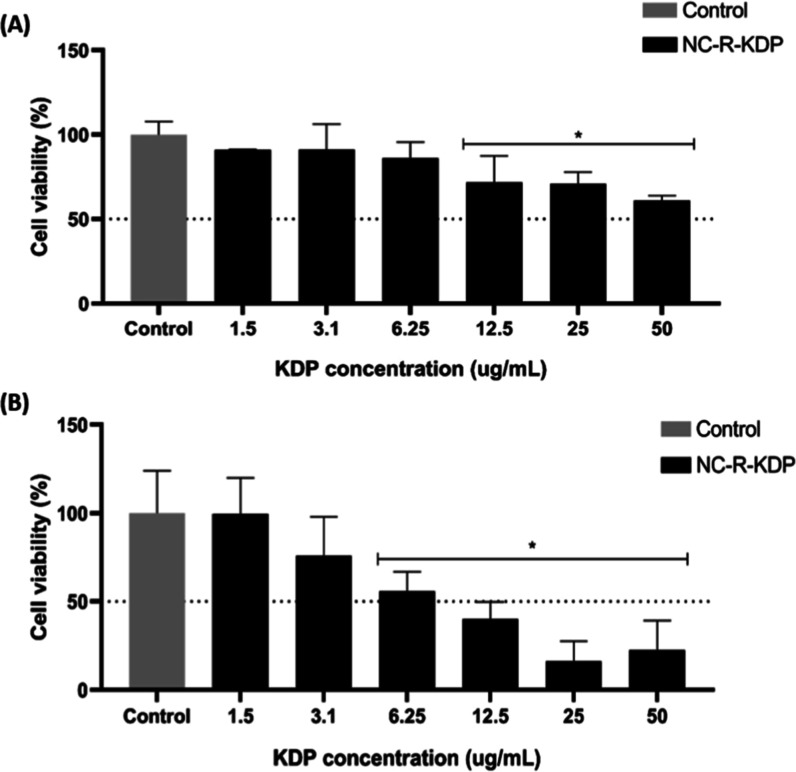
Cell viability after 24 h of treatment with KDP and rosehip oil-loaded nanocapsules (NC-R-KDP) at concentrations ranging from 1.5 to 50 μg/mL of KDP in fibroblasts (3T3-L1) (A) or normal human epidermal melanocytes (NHEM) (B). * represents a significant difference compared with the control (*p* ≤ 0.05).

In 3T3 cells (Figure 7A), up to 6.25 μg/mL of KDP did not significantly reduce cell viability (*p* > 0.05), but from 12.5 μg/mL of KDP or more, a reduction in viability was observed, suggesting dose-dependent behavior. Despite these findings, the cell viability of fibroblasts remained above 50%, even at the highest concentration tested. On the other hand, for NHEM cells (Figure 7B) the dose-dependent profile of the KDP was well observed, with higher concentrations leading to lower viability. At the highest concentrations (25 and 50 μg/mL), the cell viability decreased to less than 30%, suggesting greater sensitivity of melanocytes than fibroblasts to the formulation. The IC_50_ for NC-R-KDP in NHEM cells was calculated to be 8.922 μg/mL, confirming this increased susceptibility. It was not possible to calculate the half-maximal inhibitory concentration (IC_50_) of NC-R-KDP in fibroblasts, since 5% of the formulation did not lead to such decay in viability, which means that the IC_50_ of NC-R-KDP is greater than 50 μg/mL of KDP.

These results reflect not only a dose-dependent reduction in cell viability, which could be related to the presence of KDP or other nanocapsules components but also intrinsic differences in the sensitivity of the cell lines. 3T3 fibroblasts, an immortalized cell line, exhibited greater cell viability even at higher doses of NC-R-KDP. In contrast, NHEM melanocytes, which are primary cells derived from human tissue, exhibited greater sensitivity, an expected outcome given their physiological relevance and limited proliferation capacity. Compared with continuous cell lines, primary cells are more demanding in terms of culture conditions and more susceptible to environmental stress and exogenous substances, which can lead to lower thresholds for cytotoxic effects.^60^ Moreover, when comparing normal human dermal fibroblasts with normal human epidermal melanocytes, Kroll and co-workers (2005) reported that treatment with 4-tertiary butyl phenol, a bleaching compound, resulted in greater cell viability in fibroblasts, indicating that fibroblasts were less sensitive to the compound than were melanocytes.^61^

3T3 cells have been previously used to test the cytocompatibility of kojic monooleate (KMO) nanoemulsions.^62,63^ KMO is also an ester of kojic acid. KMO oil was compared to KMO nanoemulsion and both formulations, when the maximal concentration tested was 100 μg/mL, a 72 h treatment led to a decrease in cell viability of approximately 45%, and both formulations had an IC_50_ greater than 100 μg/mL.^63^ In the present study, we found that NC-R-KDP, when tested in 50 μg/mL of KDP, led to a decay close to 40%. Afifah and co-workers reported that the survival rate of cells treated with the KMO nanoemulsion was greater than that of cells treated with the KMO oil, suggesting safety of the nanoformulation.^63^ Similarly, Roselan and co-workers tested KMO nanoemulsion in a 24 h treatment at concentrations up to 500 μg/mL, and the IC_50_ was found to be greater than 500 μg/mL.^62^ In our previous study with a 1 mg/mL KDP nanoemulsion, the cell viability assay with 3T3 cells was employed with a 24 h treatment with up to 1% of the formulation, which led to no decrease in cell viability.^11^ In the present study, when NC-R-KDP was used at the same KDP concentration (1 mg/mL), the cytocompatibility of the formulation reached 0.625%. However, the next higher concentration tested (1.25% of the formulation) led to a reduction in cell viability. The decay observed in the present study could be related to the exposure of the cells to the treatment and to the presence of KDP.

Regarding the use of primary NHEM cells in studies with kojic acid and its derivatives, the literature is still scarce. Therefore, the present study provides insights into the behavior of primary cells after treatment with a nanoformulation containing a kojic acid derivative, highlighting the relevance of using more physiologically representative models, such as primary human cells.

The compatibility of nanosystems with viable skin cells, such as fibroblasts, is very important considering that Contri and co-workers (2016) reported that 150 nm polymeric nanocapsules can penetrate injured skin.^40^ The nanocapsules developed and tested herein had a diameter of less than 200 nm, indicating that they could penetrate the skin if the stratum corneum is injured. The size of a nanoparticle influences its toxicity, and smaller nanoparticles are more likely to cause cytotoxicity.^64^ Moreover, different cell types can have different responses after nanoparticles treatment.^64^

### 3.8 Melanin Content Assay

Melanocytes are the cells responsible for melanin synthesis.^2,4^ Therefore, NHEM cells were selected to quantify melanin after a 96 h treatment with NC-R-KDP. The formulation was tested at KDP concentrations of 1.5 μg/mL and 3.1 μg/mL, which showed cytocompatibility after 24 h treatment (Figure 7B). Additionally, cell viability remained above 80% after a 96 h treatment with NC-R-KDP at 1.5 μg/mL and 3.1 μg/mL of KDP, which was statistically equal to that of the control group (data not shown).

Treatment with NC-R-KDP at 1.5 μg/mL of KDP had no effect on melanin production (Figure 8A). However, when the treatment with the nanocapsules was increased to 3.1 μg/mL of KDP, the melanin content was significantly lower than that of the control group (*p* < 0.05) (Figure 8B). These results indicate that NC-R-KDP inhibits melanin synthesis in a dose-dependent manner in NHEM cells. It is important to mention that the cells used in the assay were derived from two different donors. Compared with those in the 1.5 μg/mL treatment group, the cells in the 3.1 μg/mL treatment group (Figure 8B) presented greater basal pigmentation and more dendritic morphology (Figure 8A), which may reflect interindividual differences in melanogenic activity and cellular responsiveness. The low basal pigmentation of the cells used for the 1.5 μg/mL treatment might have impaired the nanocapsules efficacy.

**Figure 8:**
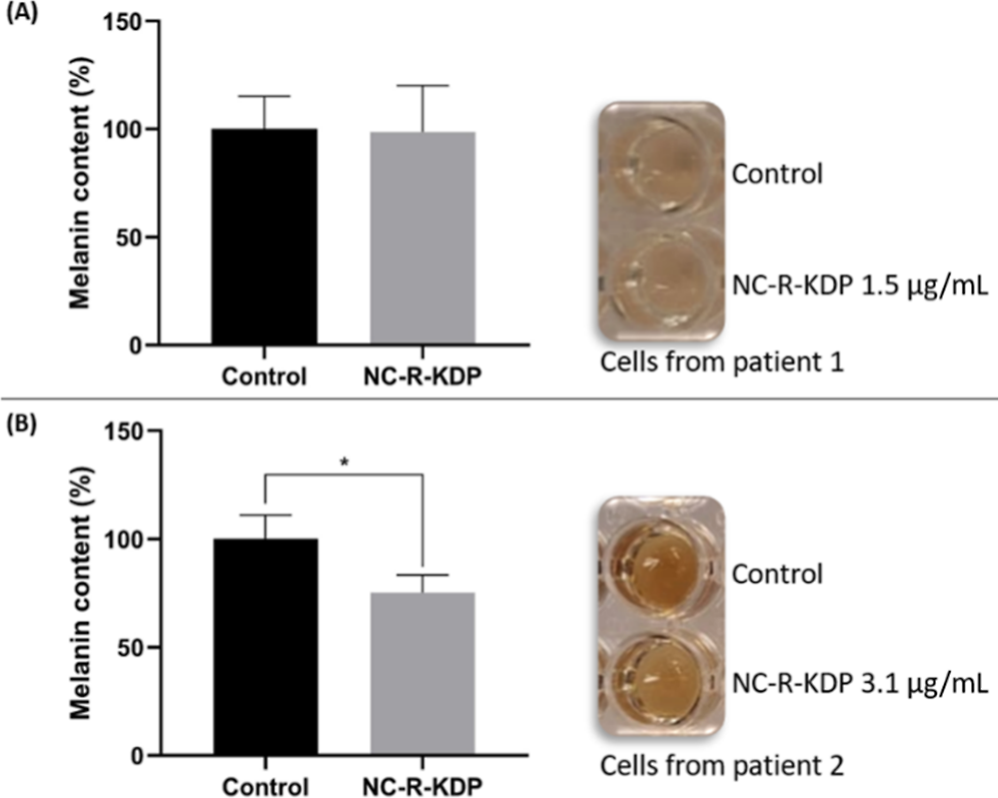
Melanin content in normal human epidermal melanocytes (NHEM) after 96 h of treatment with KDP and rosehip oil-loaded nanocapsules (NC-R-KDP) at concentrations 1.5 μg/mL of KDP (A) and 3.1 μg/mL of KDP (B). * represents a significant difference compared with the control (*p* ≤ 0.05).

Normal human epidermal melanocytes (NHEM) were used by Niki et al. (2011) to investigate the effects of 1-(2,4-dihydroxyphenyl)-3-(2,4-dimethoxy-3-methylpheny)­propane (DP), a tyrosinase inhibitor, in melanin synthesis.^65^ After 7 days of treatment, the melanin content decreased in a dose-dependent manner, with approximately 40% inhibition at 10 μM of DP. The compound exhibited an IC50 of 200 μM in the inhibition of human tyrosinase (obtained from NHEM). Kojic acid was used as a positive control to assess human tyrosinase activity, obtained from NHEM cells, and it showed a dose-dependent behavior with an IC50 of 300 μM, in contrast to its IC50 of 2 μM for melanin synthesis inhibition.^65^ Similarly, in the present study, NC-R-KDP, which contains a kojic acid derivative, also showed a dose-dependent behavior in inhibiting melanin synthesis in NHEM after a shorter exposure time (96 h).

There are a variety of studies using mouse melanoma cells from the B16 family that have demonstrated that treatment with kojic acid or its derivatives leads to a dose-dependent reduction in melanin synthesis.^66–68^ This trend was observed across different formulations, including nanotechnological formulations, and treatment durations ranging from 24 to 72 h. These findings are in accordance with those of the present study and further support the effectiveness of kojic acid derivatives in modulating melanogenesis, even when tested in primary human melanocytes such as NHEM cells.

Melanin inhibition can be obtained by diverse pathways, such as tyrosinase inhibition and antioxidant activity, and both KDP and rosehip oil nanocapsules have been demonstrated. The cellular assay performed herein corroborates the previously obtained results, further highlighting the potential of the formulation.

## 4 Conclusion

It was possible to develop polymeric nanocapsules containing kojic acid dipalmitate and rosehip oil with suitable nanoscale features. Among the four different polymers tested, PLGA stood out, maintaining the formulation’s stability throughout 180 days at refrigerated storage, leading to less degradation of KDP at room temperature. The PLGA nanocapsules allowed KDP to reach deeper skin layers, forming a stratum corneum deposit of KDP. Antioxidant activity by radical scavenging activity (DPPH assay), probably due to the presence of rose-hip oil, and by preventing the occurrence of an oxidation reaction (β-carotene/Linoleic assay), probably due to the presence of KDP, was also observed. The nanocapsules also showed ability to inhibit tyrosinase and to decrease melanin content (25% reduction when used at 3.1 μg/mL). The developed nanocapsules showed cytocompatibility for up to 0.625% of the formulation in fibroblast-like cells (3T3-L1) and 0.313% in adult, human melanocytes (NHEM cells). The formulation presented in this study shows potential for use in cosmetic formulations with skin whitening and/or lightning purposes.
